# Periscope of Dermatology

**Published:** 1898-11-05

**Authors:** 


### Periscope of Dermatology.

(*Continued from page* 84.)

**Parasitic Diseases.**—Sheffield[9] describes an easy and rapid method of curing ringworm. After clipping the hair close to the scalp the following mixture is applied: ℞. Acidi carbol. olei petrolei. āā 65 parts, tinct. iodi. olei ricini, āā 110 parts, olei rusci (German) q. s. ad 500 parts. This is applied all over the scalp, more thickly on the affected parts, with a painter's brush once a day for five successive days. On the sixth day it is wiped off with a rag dipped in plain olive oil; the hair is again clipped and the scalp washed thoroughly with green soap and a soft nail brush, care being taken to remove all the scales and loose hairs. No epilation is, as a rule, necessary. On the seventh day the mixture is reapplied as thickly as before, and the whole process is repeated regularly for three or four successive weeks—the length of time depending on the severity of the case—when it is found that the new hair has begun to grow, and no trichophyton fungi can be discovered in the hair epilated. These procedures are followed by a few days' application of a 10 per cent. sulphur ointment, and then by the use of the following preparation for about two weeks: ℞, Resorcini, acidi salicyl, āā 16 parts; alcoholis, 120 parts; olei ricini, q.s. ad 500 parts. This mixture not only hastens the growth of the hair, but prevents re-infection. Ehrmann[10] recommends the application of remedies to sycosis by means of cataphoresis. He employs the following apparatus. The cathode is formed of a glass globe provided with a rubber ring at the periphery to insure close contact, and having at the bottom an amalgamated zinc plate, the balance of the space being filled with cotton impregnated with a 10 per cent. solution of ichthyol. This cathode is applied to the diseased part, and an ordinary anode to the arm. Twelve cases of sycosis parasitica, and 37 cases of the coccal variety were treated. Nine daily sittings were required on an average in the former cases to effect a cure, and in the latter the period required for a cure ranged up to 15 months, the average time being about seven weeks; the treatment in many being applied daily, but in some only three times a week. A current of 15 milli-amperes was employed, and each affected part was treated for ten minutes at a time.

**Mycosis Fungoides.**—Castel Leredde[11] report a case of a man aged 68 years, who had suffered for five or six years with general pityriasis. A large part of the integument resembled orange peel, and on the thorax there were irregular red swellings. All the lymphatic glands were enlarged. Microscopically the lesions were found to resemble mycosis.

Whitfield[12] describes a case of mycosis fungoides occurring in a man aged 64 years. He had a rash all over him, and several sore places. He had suffered from desquamative dermatitis for four years, and one year before he had noticed a tumour on the side of his right arm. Several other swellings had appeared, which had burst and fungated, leaving ulcers. Portions of the edges of these ulcers were removed and examined bacteriologically and by inoculation on animals, with negative results. The patient died shortly afterwards.

**Drug Rashes.**—Baudouin and Emery[13] describe a case of bullous erythema over the buttocks and lower extremities, which occurred a few minutes after taking a dose of antipyrin for neuralgia. The patient was a young woman, who had suffered from two similar attacks of bullous erythema, once after eating mackerel, and the other time without any definite cause.

Hallopeau[14] describes the rôle played by toxins in the production of diseases of the skin. He divides them into exogenous, endogenous, and those of mixed origin. Exogenous toxins, or toxexogenes, may be of animal or vegetable origin; the former produce erythema, œdema, vesication, suppuration, and sphacelus. The polymorphous eruption of scabies is probably due to toxins secreted by the acarus. Rashes due to the ingestion of animal toxicogenes are more rarely observed, but urticaria following the eating of mollusks come under this head; injections of blood serum should also be included; the eruptions produced being urticarial morbiliform, or scarlatiniform. Vegetable toxicogenes may act directly upon the skin after absorption into the blood, producing erythema, urticaria, vesicles, pustules, bullæ, ecchymoses, eschars, &c. They are the result of the action of the toxins on the centres of trophic and vascular innervation. The chief vegetables producing toxins pathogenic to the skin are the trichophyton, and the parasites of actinomycoses, and of Madura foot. Endogenous toxins may be the result of the functions of the body cells, which become hurtful through excessive quantity or alteration in quality, the reabsorption of these products causing auto-intoxications, which react on the skin. Toxins produced by microbes may be considered as of mixed origin, the microbes behaving like the cells of the organism. Skin lesions may remain limited to the immediate neighbourhood of the microbic focus, or may extend centrifugally.

**Various.**—Hartzell[15] reports a case of infectious multiple gangrene of the skin which occurred in a woman aged 46 years. There were numerous perfectly round, deeply excavated, and sharply defined ulcers, and innumerable small, circular, dead white scars all over the body. Some of the ulcers looked healthy, while others were covered with greyish or black moist sloughs. The disease commenced four years before, when the patient ran a meat-hook under the nail of the third finger of the right hand; a painful spreading ulcer formed in this position, and was followed by others in distant situations; since this time she had never been free from ulcers. The ulcers commenced as pale red papules which, within a few hours, became vesicular, then pustular, and finally a slough formed. The ulcers continued to spread until destroyed by some treatment. On examining portions of the skin and edges of the ulcers, a large number of bacilli were found. If the ulcers were completely excised the wound healed readily, but if any of the base were left, the whole wound became a large gangrenous ulcer. He recommends treatment by hypodermic injections of Condy's fluid, and frequent bathing of the ulcer with 1 in 1,000 perchloride solution.

Brooke[16] is inclined to believe that zoster is an infectious disease. The following are the chief reasons for this opinion: (1) One attack confers immunity in most cases; (2) the presence of fever, and the similarity of the attack to the other exanthemata; (3) the transmission of the disease from one person to another, and the way in which several members of a family are attacked in rapid succession; (4) the occurrence in epidemics; (5) it is endemic in certain places; (6) the enlargement of the lymphatic glands, either in the neighbourhood of the eruption or on the other side, and sometimes a general adeopathy; and (7) the development of paralysis in conjunction with the eruption, and of pain, &c., in distant parts. He assumes that the infective agent in zoster attacks the sympathetic ganglia. The exact nature of this agent still remains unknown.

Winfield[17] reports the result of the post-mortem examination of a case of congenital ichthyosis, which died shortly after birth. The thyroid gland was absent, and numerous colonies of micrococci were found in the lymph spaces of the skin. He considers that the micrococci were the result of intra-uterine infection.

[9] New York Med. Jour., May 14, 1898, p. 680. [10] Amer. Med. Surg. Bull, Mar. 25, 1898, p. 267. [11] La Semaine Med., Mar. 16, 1898. [12] Brit. Jour. of Derm., May, 1898, p. 133. [13] La Semaine Med., Mar. 18, 1898. [14] Ann. de Derm. Syph., 1898, Nos. 8 and 9; Amer. Jour. Med. Sci., Feb, 1898, p. 233. [15] Ditto, July, 1898, p. 43. [16] Med. Chron., April, 1898, p. 67. [17] Amer. Med. Surg. Bull., April 10, 1898, p. 319.

